# The progress of *Mycobacterium tuberculosis* drug targets

**DOI:** 10.3389/fmed.2024.1455715

**Published:** 2024-10-21

**Authors:** Xin Zhang, Ruixia Zhao, Yao Qi, Xiong Yan, Gaoxiu Qi, Qiuju Peng

**Affiliations:** ^1^Qingdao Central Hospital, University of Health and Rehabilitation Sciences, Qingdao Central Medical Group, Qingdao, Shandong, China; ^2^Qingdao Chest Hospital, Qingdao, Shandong, China

**Keywords:** *Mycobacterium tuberculosis*, anti-tuberculosis drug, target, cell wall synthesis correlation, DNA/RNA synthesis correlation, energy metabolism

## Abstract

Tuberculosis (TB) has been troubling humans for hundreds of years, is a highly infectious disease caused by *Mycobacterium tuberculosis* (Mtb) infection, Mtb can infect almost all organs of the body and is one of the deadly infectious diseases in the world. At present, the first-line treatment regimen has a long treatment cycle and is prone to multiple drug resistance. Anti-tuberculosis drugs and latent tuberculosis infection (LTBI) resistance are increasing year by year, and new targets and new bioactive compounds are urgently needed to treat this disease. This review focuses on the latest reported anti-TB drug targets and related compounds in recent years, reviews the current TB drug regimen and major defects, outlines the key drug targets developed to date in Mtb, and the current situation of newly discovered anti-TB resistant forms of drugs. To provide a reference for the research and development of new anti-TB drugs and bring new treatment strategies for TB patients.

## 1 Introduction

Tuberculosis (TB) is caused by *Mycobacterium tuberculosis* (Mtb) infection and has plagued humans for centuries. Mtb is transmitted primarily through aerosols excreted by humans with active TB. Mtb can cause TB in any part of the body, especially in the lungs. After infecting the host, Mtb enters the lungs and is engulfed by macrophages. Immune cells aggregate and block infected macrophages, gradually forming granulomas that include Mtb, the hallmark of TB (1). This also leads to inapparent infection, at which stage the infection is controlled, but it is easily at risk of reactivation. A significant proportion of the population is infected with latent tuberculosis infection (LTBI) (2, 3). LTBI is asymptomatic and noncontagious and remains controlled in most individuals. 25% of the global population is infected with latent tuberculosis (LTBI) (4). However, some people develop active TB when their resistance is reduced.

In 2022, the WHO found that about 10 million new active TB patients and 1.6 million people die of TB each year worldwide (5). Tuberculosis (TB) remains one of the major global health problem (6). Since the emergence of COVID-19, the number of TB patients reported to clinics has decreased globally, with some developing countries reporting 25–30% reductions in TB cases (7). The global reduction in TB treatment by more than 1 million has reportedly set back the fight against TB by a decade and the 2035 target for a clean bill of TB will be missed. COVID-19 can occur at any time during the progression of tuberculosis, and patients affected by active tuberculosis have a worse prognosis. The pathogenesis of both infectious agents involves cell-mediated immunity (3). Because the host immune system is weakened, one infection may increase the likelihood that the other will contract. COVID-19 and treatment with corticosteroids can also lead to active tuberculosis due to reactivation or infection with Mtb (8). More evidence is needed to understand the interrelationship between COVID-19 and TB. The COVID-19 pandemic has had a devastating impact on TB diagnosis, treatment and the disease burden of TB globally. The need for new treatment strategies to reduce the impact of COVID-19 is a new challenge in the prevention and treatment of TB (9).

Early diagnosis and prompt treatment of TB are essential to prevent further spread of the bacteria and the development of resistant strains. Effective anti-TB regimens can kill Mtb, improve clinical symptoms, and prevent further progression of the disease. Although there are a number of clinical drugs available to prevent and treat TB, their major drawback is the longer duration of use (6–9 months) for drug-susceptible TB (DS-TB) compared to up to 2 years for drug-resistant TB (10, 11). Factors such as poor patient compliance, increased drug resistance and HIV-TB co-infection lead to treatment failure and disease recurrence. New anti-TB drugs that are superior to existing treatment regimens in efficacy, tolerability, and duration of treatment are urgently needed to cure and curb the spread of TB.

## 2 Current status of anti-TB drugs

The US Food and Drug Administration (FDA) has approved 10 drugs to treat TB (12). WHO recommended in 2020 that the first-line treatment include 2 months of Rifampin (RIF), Isoniazid (INH), pyrazinamide (PYZ), and ethambutol (EMB) and 4 months of intensive RIF and INH medication. This protocol is used to treat drug-sensitive TB and represents approximately 95% of all active TB worldwide (Table 1) (10). Second-line and third-line anti-TB drugs; such as streptomycin, kanamycin, and clofazimine, are recommended for drug-resistant TB. In recent decades, only three new anti-TB drugs have been approved by the FDA, Betaquoline (TMC207 or R207910), Delamanid (OPC67683), and Pretomanid (PA-824) (Table 2) (13). The side effects of TB drugs also lead to poor patient compliance, difficulty in tolerating, and spontaneous drug withdrawal, which further promotes the enhancement of drug resistance of Mtb. Mtb exhibits differential resistance in different individuals, so how to select drugs remains one of the challenges facing clinicians.

**Table 1:** Mechanism of action of standard first line anti-TB drugs and their aside effects.

Drug discovery	Date	Target	Mechanism	Description	Potency	Side effect
Rifampicin (RIF)	1963	Bacterial RNA polymerase (RNAP)	Inhibits bacterial DNA-dependent RNA polymerase Broad antibacterial spectrum, including activity against several forms of Mycobacterium.	Rifamycin group of antibiotics	Bactericidal	Hepatitis is the most, joint pain, fever, headache
Isoniazid (INH)	1952	InhA[Enoyl-(acyl-carrier-protein) reductase]	Inhibits mycolic acid biosynthesis by Cell envelope disruption	Pyridine-carboxylic class of drugs, containing a pyridine ring bearing a carboxylic acid group	Bactericidal	Psychiatric disorders restlessness, insomnia, muscle twitching
Pyrazinamide (PYZ)	1952	S1 Component of 30S Ribosomal subunit	Acidifies cytoplasm; Inhibits translation and trans-translation, disrupt membrane energetics and inhibit membrane transport function at acid pH	Z-A pyrazine based compound active against tubercle bacilli in acidic inflammatory lesions	Bactericidal	Elevated uric acid, gastrointestinal upsets anorexia, arthralgia, gout
Ethambutol (EMB)	1961	Inhibits arbinosyl- transferase	Ethambutol disrupts arabinogalactan synthesis to Cell wall disruption	E-1,2-Aminoalcohols	Bacteriostatic	Peripheral neuritis and Optic neuritis

**Table 2:** Mechanism of action of new anti-TB drugs and their aside effects.

Drug discovery	Date	Target	Mechanism	Description	Potency	Side effect
Betaquoline (TMC207 or R207910)	2012	ATP synthase	Inhibits ATP synthesis by targeting mycobacterial ATP synthase	(Diaryl) quinoline	Bacteriostatic	Nausea and headache and QTc prolongation
Delamanid (OPC67683)	2014	Mycolic acid	Inhibits mycolic acid synthesis and cellular respiration	Nitro-dihydro-imidazooxazole derivative	Bactericidal	Cause QTc prolongation, anorexia, gastritis, malaise, anemia, and psychiatric disorders
Pretomanid (PA-824)	2019	–	PA inhibits mycolic acid synthesis, a necessary step in cell wall formation	Nitro-dihydro-imidazooxazole derivative	Bactericidal	Gastrointestinal symptoms, vomiting and transaminase increase, hepatotoxicity, and headache

However, due to its many use conditions and the limitation of easy adverse reactions, it cannot fundamentally solve the shortage of anti-TB drugs. This situation encourages researchers to have a deep understanding of the physiological properties and pathogenic mechanisms of Mtb, to discover new anti-TB targets and further develop new anti-TB drugs.

## 3 Anti-tuberculosis drug targets

Whole-genome sequence sequencing of Mtb provides a platform for a wide range of novel drug targets and regimens for anti-TB therapy. Mtb comprises 4,411,529, base pairs, contains approximately 4,000 genes, and has a very high guanine + cytosine content, which is reflected in the biased amino acid content of the protein (14). Based on target and phenotype-based methods have been used to find these targets of inhibitors, combined with computer-aided drug design (CADD) support, based on quantum mechanics and molecular simulation theory, through simulation, calculation, or model learning, predict the role between drug and target molecules, screening, design and optimization of the lead compound (15, 16). Antimicrobial drugs and compounds in clinical trials are mostly synthesized for macromolecules (i.e., cell walls, proteins, and nucleic acids), providing an attractive direction for the future discovery of anti-TB drugs.

### 3.1 Cell wall synthesis correlation

**Figure 1:**
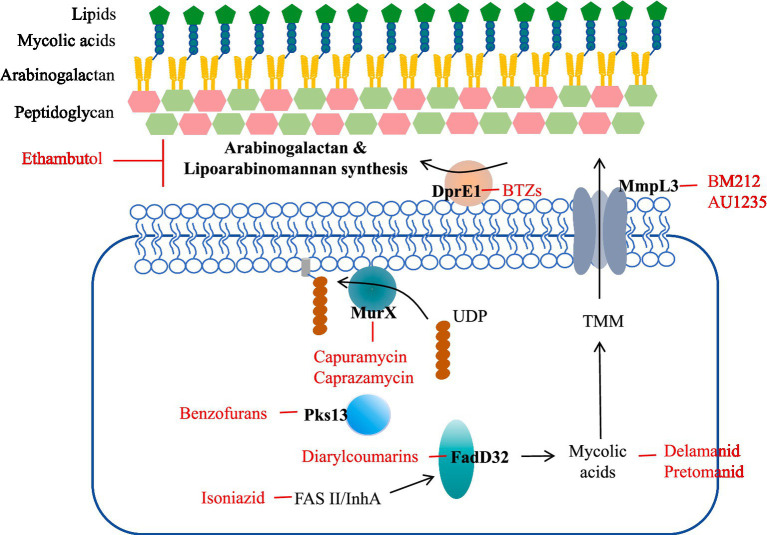
Mtb cell wall structure and targets as well as examples of compounds that inhibit them. Front-line drugs (isoniazid, ethambutol) are shown in purple.

The cell wall is a barrier to the internal and external environment of the cell and is involved in many fundamental processes of biological metabolism, including structure definition, protection, and transport (17). The cell wall of Mtb is associated with its pathogenicity and invasion, and cell wall biosynthesis is a critical antibiotic target (18). The cell wall of Mtb consists of three main structures: an outer mycolic acid (MA) layer, an arabinogalacto-polysaccharide (AGP) intermediate layer, and an inner peptidoglycan (PG) layer (Figure 1) (19). Peptidoglycan (PG) layer is highly cross-linked surrounding the plasma membrane and forms a covalent complex with Arabinogalactan (AGP) (20). PG tissue is dynamic, maintaining cell wall thickness and acting as a physical barrier to protect bacteria from potential damage in the microenvironment. The length of AGP polysaccharide affects the shape and hydrophobicity of the membrane. The superficial AG ends are esterified with abnormally long high-molecular-weight fatty acids called mycolic acids, which represent a major part of the cell wall. Mycolic acid is highly hydrophobic and forms a shell around the bacterium to resist hydrophilic antibiotics, oxidative damage, and complement deposition. All three of these molecular entities can serve as targets for inhibition (21).

Targeting the mycobacterial cell wall is an effective strategy, such as two first-line antitubercular drugs, INH and ethambutol, and several second-line drugs, including ethionamide, thiolactone, cycloserine, and terizidone. Many families of new drugs have been associated with the construction of cell wall components through whole-cell screens targeting essential proteins.

#### 3.1.1 DprE (decaprenylphosphoryl-D-ribose 2′-epimerase)

**Figure 2:**
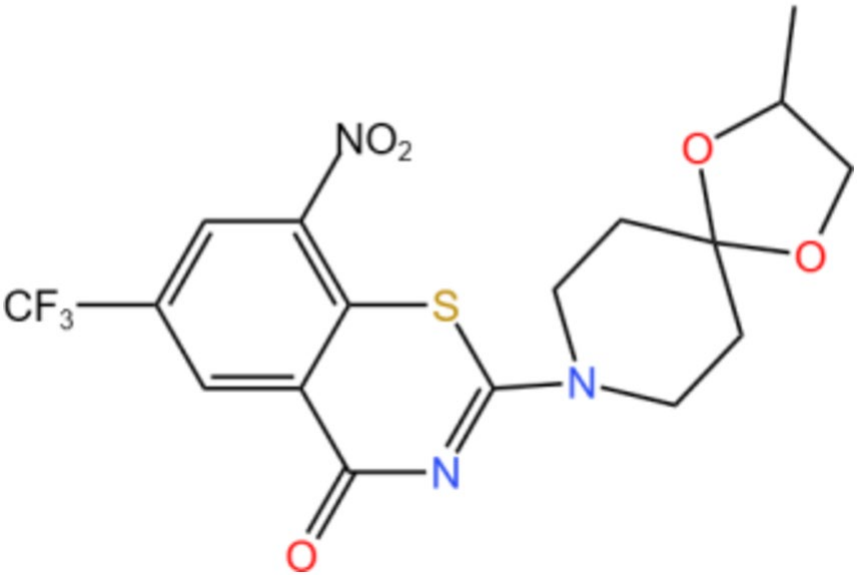
Structure of DprE1.

Decaprenylphosphoryl-*β*-D-ribose-2′-epimerase (DprE1; EC 1.1.98.3) is a widely investigated target in mycobacteria species. DprE1 is an indispensable flavoenzyme involved in forming the Mtb cell wall. It is a heterodimer composed of DprE 1 and DprE 2, the key enzyme catalyzing the conversion of decaprenyl-phospho-ribose (DPR) into D-ribose to D-arabinofuranose (decaprenyl-phosphoryl D-arabinose, DPA), and DPA is the only precursor for the synthesis of cell wall arabinoglycan (Figure 2) (22, 23). DprE 1 thus becomes a promising target for the development of novel drug candidates to treat TB. Have identified 23 new classes of DprE 1 inhibitors that demonstrated anti-mycobacterial activity and their different scaffolds. The mechanism of action is mainly divided into two categories, including covalent binders and non-covalent inhibitors (24, 25). The covalent binder produces covalent adducts that irreversibly inhibit DprE1 with C387 residues, and non-covalent inhibitors act as competitive inhibitors (26).

DprE-type 1 was first identified as a target of benzothiazone and its mechanism of action is nitro-dependent, through irreversible inhibition of DprE 1 through the generation of covalent adducts with the amino acid Cys387 (27). Makarov’s team demonstrated for the first time that benzothiazole (BTZ) strongly inhibits DprE 1 activity *in vitro* and *in vivo* (28). So far, more than 600 new nitrobenzothiazinones have been studied, and nearly 90% of these molecules are active against Mtb (MIC <10 *μ* M) (29). In anti-TB studies, some pyrimidine derivatives are mainly substituted pyrimidine nucleosides that inhibit thymine monophosphate kinases. Derivatives containing the pyrimidine cores in the three constructs are currently being investigated in clinical trials. Among them, the nitrogen derivative TBA-7371 is a DprE1 inhibitor in phase 2 dose-escalation trials to assess safety, early bactericidal activity (EBA), and pharmacokinetics (NCT04176250) (30). Kb et al. (31) screened azido derivatives using a structure-based pharmacophore model and further verified by induced matching docking and molecular dynamics combined with Prime MM-GBSA calculation, selected the two compounds from the ZINC database and compared the results with the inhibitors of clinical trials, yielding a compound with the greatest inhibitory potential for DprE 1.

#### 3.1.2 Mycobacterial membrane protein large 3

**Figure 3:**
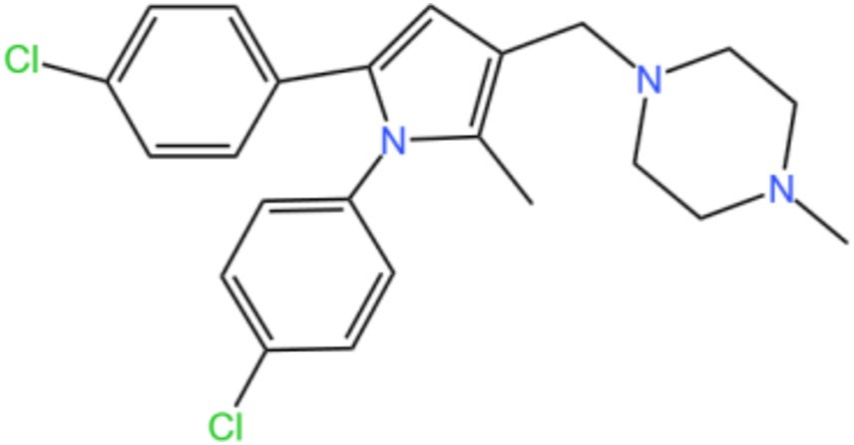
Structure of MMPL3.

**Figure 4:**
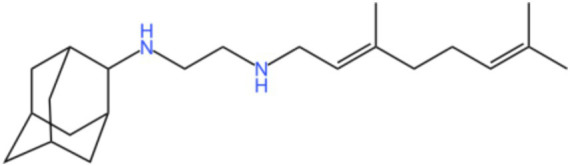
Structure of SQ109.

Mycobacterial membrane protein large 3 (MmpL 3) is a transmembrane transporter protein, mainly involved in the lipid transport, polymerization, and immune regulation of Mtb, and is an important drug target for anti-TB therapy (Figure 3) (32). MmpL 3 is an inner membrane transporter necessary for constructing the mycobacterial outer membrane, and is involved in mycolic acid synthesis, the main component of the mycobacterial outer membrane. Very susceptible to chemical inhibition, and the large number of chemical scaffolds identified by whole-cell phenotyping screening can kill mycobacteria by inhibiting this transporter (33). MmpL 3 inhibition impairs the Mtb cell wall and guides cell death. Currently, there is no FDA-approved TB treatment drug for the MmpL 3 receptor. The molecules that inhibit MmpL 3 have no common pharmacophore and have different scaffolding effects (34). Various newer classes of compounds, such as in-2-carboxamide, pyrrole, pyrazole, benzimidazole, benzimidazole, spiral ring, piperidol, benzothiazolamide, and aminamide urea have been reported to act as MmpL 3 inhibitors. Of all the MmpL 3 inhibitors, SQ 109 is the most clinically advanced, and SQ 109 is an analog of EMB (Figure 4), a well-established MmpL 3 inhibitor with completed phase 2b-3 clinical trials (35). SQ 109 was well tolerated in humans, and a phase IIb study found that it was significantly with rifampicin (36). Low doses of SQ 109 showed significant synergies with first-line drugs (INH and RIF) and aquiline. These combinations also reduced the treatment time by 25 to 30% (37). The metabolism of SQ 109 occurs mainly in the oxidized form at many positions in the adamantane ring, containing a 1-adamantane group and a geranyl group attached to two nitrogen atoms, which form the core of ethylenediamine. EMB is a type of ethylenediamine and, similarly to SQ 109, EMB is a cell wall inhibitor, however, EMB inhibits the formation of the arabinogalactan layer, rather than Mmpl 3-mediated mycolic acid transfer (38). The placement of EMB with SQ 109 in TB treatment also showed higher efficacy and shorter treatment time. This feature makes SQ 109 a unique drug and an excellent component of combination therapy for TB. SQ 109 is expected to be an excellent anti-TB agent.

#### 3.1.3 L-rhamnose synthesis-related enzymes

L-Rhamnose is a deoxyhexose that is one of the important cell wall components of Mtb. Since there is no L-rhamnose component in humans, its synthesis-related enzymes become alternative drug targets. The structural integrity of the cell wall based on l-rhamnose ligation plays an important role in maintaining the viability of Mtb. The dTDP-L-rhamnose is synthesized from glucose-1-phosphate (G1P) and deoxythymidine triphosphate (dTTP) by four enzymes, The enzymes involved in the reaction are Rml A (glucose-L-phosphate thymidylyltransferase), Rml B (dTDP-glucose-4, 6-dehydratase), Rml C (dTDP-6-deoxy-D-xylo-4-hexulose-3, 5-epimerase) and Rml D (dTDP-6-deoxy-L-lyxo-4-hexulose C4-reductase). Rml A is the rate-limiting enzyme in this pathway. Qu et al. (39, 40) showed that Rml A knockdown can affect cell growth and morphology in Mtb.Rml A is a potential target for anti-mycobacterial drug development. Dhaked Through protein structure modeling and analysis of the four enzymes, dozens of potential functional residues including G8, Q80, and G85 were selected to explore the catalytic mechanism, to provide a reference direction for the future synthesis of related compounds (41). The l-rhamnose provides a powerful basis for developing anti-mycobacterial drugs to treat TB. All enzymes in the rhamnose biosynthetic pathway have been identified as promising drug targets.

#### 3.1.4 Enenyl-carrier protein reductase

Enenyl carrier protein reductase (InhA) is a tetrameric structural enzyme of TB, a key enzyme involved in fatty acid synthesis, mainly involved in mycolic acid biosynthesis, and is a part of the NADH-dependent acyl carrier protein reductase family (42). InhA can be targeted as anti-TB drugs, including indirect inhibitors (Inh, ethionicotinamide, prothionamide) and direct inhibitors (triclosan/diphenyl ether, pyrrolidine formamide, pyrrole, acetamide, thiadiazole, triazole) (43). Inh belongs to a class of precursor drugs that rely on the KatG enzyme to activate it, and genetic mutations in KatG block this activation pathway and cause drug resistance. So many studies have now focused on the “direct inhibitors” of InhA. The fraction that directly suppresses InhA activity without affecting KatG activity has been reported as a prospective drug against TB (44). Many InhA preparations have entered different stages of clinical trials, showing promising therapeutic results. InhA catalyzes the specific reduction of the fatty acid adenine enyl carrier protein (NAD +) -dinucleotide (ACP) during the elongation cycle of the fatty acid synthase (FAS) -II pathway. This NAD + −specific enzyme resolves the trans-double bond connecting the fatty acid acyl chain and the ACP at sites C2 and C3. Milate is an important component of the mycobacterial cell wall, and its bioformation is stopped by inhibition of InhA activity. Therefore, the first-line drug INH and other promising therapeutic agents have identified Mtb InhA as its primary target.

#### 3.1.5 D-alanine-D-alanine ligase a

**Figure 5:**
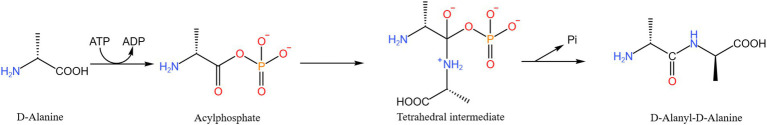
A series of chemical reactions catalyzed by DdlA.

D-alanine-D-alanine ligase A (D-Alanyl-D-alanine ligase A, DdlA) catalyzes the dimerization of two kinds of D-alanine and promotes the dimerization of D-alanine (Ala) molecules required for the biosynthesis of mycobacterial peptidoglycan mediated by ATP to obtain D-alanine-D-Ala, which can significantly affect the normal function of Mtb cell wall, and is a promising anti-mycobacterial drug target (45). The antibiotic D-cycloserine (DCS) targets the peptidoglycan biosynthetic enzyme D-Ala-D-Ala ligase (Ddl), and DCS suppresses Ddl by competitive and reversible binding to D-Ala binding sites. A cell model of *M. smegmatis* in which it is downregulated in DdlA by Sm-ddlA antisense RNA was constructed (Figure 5) by Yang et al. (46). Based on this model, DdlA deficiency was found to stimulate the increase of Z ring number and the rearrangement of cell contents with hyaline nucleoid and filamentous DNA, which further inhibited mycobacterial cell division. Proteomic analysis identified 20 differentially expressed proteins, 6 were significantly up-regulated and 14 were significantly downregulated. And proposed that DdlA deficiency results in reduced carbohydrate catabolism and inhibition of fatty acid anabolism. In the absence of DdlA, both protein degradation and synthesis remain active. Bioinformatics analysis showed that DdlA deficiency attenuated carbohydrate catabolic processes and fatty acid anabolic pathways while maintaining the degradation and synthesis of active proteins. The team showed that NDMA-dependent methanol dehydrogenase (MSMEG _ 6,242) and fumonisin (MSMEG _ 1,419) are the most differentially expressed proteins; these proteins have great potential to be developed as anti-TB drug targets (47).

### 3.2 DNA/RNA synthesis correlation

#### 3.2.1 DNA gyrase

DNA gyrase, a type II topoisomerase not present in the human body and present in Mtb, is an ATP-dependent enzyme essential in DNA transcription, replication, and chromosome segregation (48). DNA gyrase is a tetrameric protein that is composed of two GyrA and two GyrB subunits. DNA gyrase introduces negative supercoils by breaking two strands of a stretch of DNA (G-DNA), leading to strand separation. Another DNA fragment (T-DNA) catalyzes the negative supercoiling of DNA in an ATP-dependent manner. DNA gyrase is highly conserved among bacterial pathogens, and inhibition of its function can affect overall bacterial gene expression and affect bacterial functional genomics (49). Genomics studies suggest that DNA gyrase is one of the top 51 potential drug targets in Mtb. Fluoroquinolones are commonly used and effective antimicrobial agents that target DNA gyrase, however, side effects and resistance due to fluoroquinolone abuse have driven intensive research in this field. Cycloloxacin resistance due to mutations in the gyrA gene. Resistance to fluoroquinolones is usually caused by substitutions of Ser 91 and Asp 94 in Mtb gyrase A, and drug studies and target screening have been directed to the GyrB subunit. Several different classes of antibiotics are known to bind at different sites of DNA gyrase to inhibit or reduce bacterial activity for the treatment of bacterial infections. Multiple novel compounds interact with different binding sites, acting as DNA gyrase inhibitors, and some are in phase II of drug development. However, only two major sites: the ATP enzyme site and the quinolone-binding site are targets for FDA-approved drugs (50). Pakamwong et al. (51) in the Specs compound library,^1^ 30 candidate inhibitors of gyrase ATP enzyme activity of Mtb DNA were virtually selected and verified by bioassay. Two of these compounds, G24 and G26, inhibited the growth of Mtb H 37 Rv at a minimum inhibitory concentration of 12.5 μg/mL. Both compounds inhibited DNA gyrase ATP enzyme activity by IC with 50 values of 2.69 and 2.46 μ M, respectively, indicating that this is a possible basis for their anti-TB activity. The model of compounds G24 and G26 complexes generated by molecular dynamics simulations and pharmacophore map analysis binding to the Mtb DNA gyrase-ATP binding site revealed hydrophobic interactions of the inhibitor hydrophobic headgroups and electrostatic and hydrogen bonding interactions of the polar tails, which may be important for their inhibition. Reducing the compound lipophilicity by increasing the polarity of these tails thus provides a possible route for improved solubility and activity. The results show that compounds G24 and G26 target DNA gyrase, by inhibiting their catalytic cycle and thus mycobacterial growth, providing a theoretical basis for novel drug optimization that can be used in resistant strains (51).

#### 3.2.2 RNA polymerase

RNA polymerase (RNAP) plays an important role in transcription regulation. Its subunit sequence is highly conserved and is different from the structure in eukaryotes. The mycobacterial RNAP core enzyme, like other bacterial RNAP, has five subunits: the enzyme nucleus is mainly composed of *α* I and α II homodimeric *β*, β ′, and *ω*, encoded by rpoA, rpoB, rpoC, and rpoZ genes, respectively. RIF is a circular semi-synthetic antimicrobial agent derived from rifamycin. RIF targets the RpoB protein of Mtb-RNAP (the β-subunit of RNA polymerase), thus preventing the elongation of 2–3 nucleotide-long RNA transcripts by spatial blocking, and inhibiting RNA transcription and synthesis, thus effectively capturing the abortive transcriptional state and achieving bactericidal effect. Mtb strains have missense mutations in the RIF binding region [commonly called the RIF resistance determining zone (RRDR)] that affect inhibitor specificity and affinity, leading to drug resistance (52). RIF drug resistance is mainly (> 95%) and mutations in the 81 bp RIF drug resistance determining region (RRDR) of the rpoB protein gene, so the study on RRDR has become a hot topic in recent years (53). At the same time, it can identify the molecular detection methods associated with drug resistance mutations, which can further carry out the study of drug resistance mutations and guide the clinical medication. Persing et al. (85) developed the Xpert Mtb / RIF Ultra test (Ultra) to improve the performance of the bedside test for TB (TB) and rifampicin resistance (RIF-R) rapid test, and all the tested RIF-R-associated Mtb rpoB mutations were identified by Ultra. Testing the clinical sputum samples, the overall sensitivity of Ultra and Xpert was 87.5%, respectively (54). Lokuil et al. (55) developed a novel fluorescent oligonucleotide rpoBMTC probe (5 ′ -Alexa-555-AGCGGGGTGATGTCAACCCAG-3 ′) for the rpoB gene of the Mtb complex. In the computer alignment, 100% matched, and 66.6 to 47.6% for other bacteria (55). The rpoB subunits have also become a research focus for targeted therapy. Mann et al. (56) demonstrated that phenylalanine amide MMV688845 can target the rpoB subunit of RNA polymerase in Mtb, has extensive activity against *M. abscessus* complex, be bactericidal against *M. abscessus in vitro*, and shows additivity with commonly used *M. abscessus* antibiotics in the macrophage infection model, and synergistic effect with macrolide drugs (56).

#### 3.2.3 Thymidylate kinase

Mtb thymidylate kinase (TMK) is a nucleoside monophosphate kinase, the last specific enzyme in the biosynthesis of thymidine triphosphate biosynthesis. It plays a bond role in pyrimidine synthesis, is essential for the DNA synthesis of bacteria, and is essential for the growth and survival of mycobacteria (57). TMK catalyzes the reversible phosphorylation between ATP and deoxythymidine-5 ′ -monophosphate (dTMP) to produce ADP and deoxythymidine-5 ′ -diphosphate (dTDP), and again by phosphorylation to obtain thymidine 5 ′ -triphosphate (dTTP). The low homology of Mtb-TMK in the human body and its critical role in DNA replication make MtbTMPK a promising target for novel bioactive molecules for the treatment of TB. El-Shoukrofy et al. (86) designed and synthesized two series of new tetrahydropyrimidine (THPM) -1,2,3-triazole clubbed compounds, showing an inhibitory activity comparable to the natural substrate deoxythymidine monophosphate (dTMP) (58). Using computer technology, Venugopala et al. (58) identified 3-cyanopyridones and 1.6-naphthyridin-2-ones as an Mtb-TMK inhibitor and showed whole-cell antiTB activity against H 37 Rv and MDR strains of Mtb. The success of these studies will provide a better understanding of the pharmacability of thymidylate kinase and help to refine future drug design studies on this attractive drug target of Mtb.

### 3.3 Energy metabolism

All living organisms require energy to maintain their viability. In the process of finding new TB, the researchers have deeply studied the energy metabolism of mycobacteria as a novel drug target. Mtb Oxidative phosphorylation (OxPhos) and transmembrane electrochemical gradients are promising areas of research for future anti-TB drugs. Mtb as an obligate aerobic bacterium adopts an electron transport chain that includes two terminal oxidases reducing oxygen to form water, namely the cytochrome bcc/aa 3 supercomplex and cytochrome bd. During OxPhos, electrons transfer from electron donors generated in the central metabolic pathway to molecular oxygen via the ETC. The energy released during this process is conserved by proton-pumped transmembrane proteins that establish a proton gradient to create an electrochemical gradient known as the proton motive force (PMF). This bioenergetic pathway generates ATP through the phosphorylation of ADP.

#### 3.3.1 ATP-synthesizing enzyme

In Mtb, energy production occurs in two ways, OxPhos and phosphorylation at the substrate level. Mtb is an obligate aerobic bacterium that makes it dependent on OxPhos for ATP synthesis and growth. ATP is produced by ATP synthase, leading to the growth and proliferation of mycobacteria. ATP synthase in Mtb is a transmembrane protein complex with multiple subunits, consisting mainly of transmembrane parts (F0 and subunits A, b, and c) in which subunit c forms a circular structure and a cytoplasmic part (F1 with subunits *α*, *β*, *γ*, *δ* and *ε*) (59). ATP synthesis is due to the passage of the proton through f0, triggering the rotation of the oligomeric subunit c, which is coupled to the rotation of the (α β) 3 hexamer of F1. Betaquinoline (BDQ) inhibits mycobacterial ATP synthase, a heteropolymeric complex of two subunits, but it does not interfere with mammalian ATP synthase. Bettaquilin has become the drug of choice for MDR-TB and contributes to shortening treatment time. The main target of beta quinoline is the C subunit of ATP synthase encoded by the atpE gene. However, bedaquinoline causes a series of adverse reactions in patients, including arrhythmia, hepatotoxicity, nausea and vomiting, and headache, limiting its clinical application. New therapeutic alternatives are urgently needed. By developing analogs of BDQ, Dhulap et al. (59) found that diarylquinoline, TBAJ-587, and TBAJ-876 compounds showed potential efficacy against TB with high clearance, reduced lipophilicity, and reduced threat of QT extension, and both classes of compounds are in the first stage of clinical studies. The biological activity of imidazolopyridine ether derivatives was studied. The results indicate that a benzene ring of [1,2-A] structures attached to imidazole and nitrogen is an essential pharmacophore. The compound inhibited ATP synthase by 8-fold. Shahul Hameed P Two different lead series of imidazopropyridine ether (IPE) and tetonamide (SQA) are reported as inhibitors of mycobacterial ATP synthesis. SQAs were found to be noncytotoxic and showed efficacy in a mouse model of TB infection (60). There are many other types of ATP synthase inhibitors, such as a non-diarylquinoline scaffold 6,7-dihydropyrazolo [1,5-A] pyrazin-4-one28587823, sulphonamide derivatives (61, 62). ATP synthase inhibitors may be an alternative to TB.

#### 3.3.2 Cytochrome bc 1: aa 3

Mtb has two terminal oxidases in its respiratory chain: cytochrome bcc 1/aa 3 (cyt-bcc 1/aa 3) supercomplex and cytochrome bd oxidase (cytbd) (63). Cytochrome bc 1: aa 3 of Mtb is a complex composed of menadione, cytochrome c reductase (bc 1), and cytochrome aa 3-type oxidase, which mainly exerts electron transport efficacy in the respiratory chain. In the absence of stress, cyt-bcc 1/aa 3, as the main terminal oxidase of Mtb, produces a large amount of ATP (64). Under stress conditions such as the hypoxic state, nutrient deficiency, and unfavorable environmental states, cyt-bcc 1 / aa 3 is normally inhibited and Mtb enters a non-replicative dormant state. Imidazolopyridine amine telacebec (Q203), a pyridine derivative that blocks the cytochrome b subunit (the cytochrome b subunit, QcrB) in the cytochrome bc 1: aa 3 complex, and effectively inhibits the growth of TB bacilli (65). However, the cytochrome bd oxidase in the respiratory chain can compensate for ATP synthesis and thus guarantee the survival of bacteria. Thus although Q203 showed a good response before phase II of the clinical trial, it only produced a bacteriostatic response without not sterilization. The introduction of cyt-bd inhibitors into the drug cluster should be a sensible approach to ensure an inhibitory response to drug-resistant TB strains. To effectively close mycobacterial energy conversion and to kill Mtb, targeting both branches of the respiratory chain may be required. Lu et al. (66) found that inactivation of cytochrome bd by small molecule inhibitors or gene modification could convert the bacteriostatic activity of Q203 into bactericidal activity (66).

**Figure 6:**
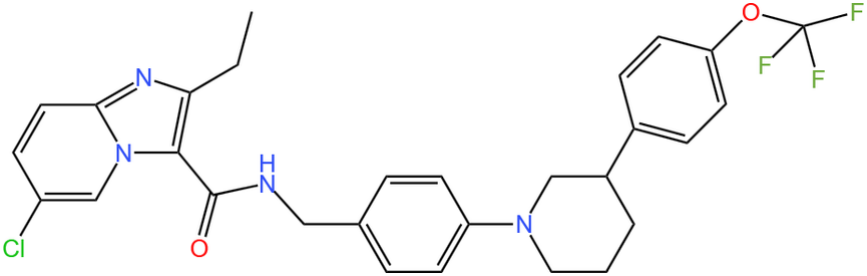
Structure of Q203.

Kalia et al. (67) used genetic and chemical biology methods to knock down the gene encoding cytochrome bd oxidase cydAB, Q203 completely inhibits mycobacterial respiration, has a bactericidal effect, kills drug-resistant mycobacteria persistence, and rapidly eliminates Mtb infection in the body (Figure 6) (67). The results show that two terminal respiratory oxidases complement each other, and simultaneous inhibition of both terminal oxidases can stop respiration, kill non-replication-resistant Mtb, and eradicate *in vivo* infection at an extremely rapid rate, which can be used for anti-TB drug development. Chilamakuru et al. (68) through comprehensive molecular docking study obtained a series of unique mycobacterial cytochrome bc 1 complex inhibitors: naphthoquinone pestle butanone scaffold, series-2,2a, 2c, 2 g, 2 h, and 2j of five compounds showed significant *in vitro* anti-TB activity against MtbH, these synergistic studies with INH and RIF showed that compound 2a at a concentration of 0.39 μg/mL (68). The combination of bacteriostatic agents and fungicides can effectively kill Mtb, laying a foundation for the research of combination therapy.

#### 3.3.3 Cytochrome P450 enzyme line

Cytochrome P450 (CYPs) is CYP and heme-containing monooxygenase, a class of redoreductase with mixed functions. It plays a key role in detoxification, cell metabolism, and homeostasis, and is an essential key enzyme for cell synthesis and catabolic activities (69). The 4.4 Mb genome of Mtb H37Rv contains 20 CYP genes as found by Cole et al. (14). The high abundance of the CYP gene is a general feature of mycobacteria, making it a potential target for anti-TB therapy. Bukhdruker et al. (87) determined that Mtb CYP was able to biotransform the antitubercular drug SQ 109, demonstrating that the Mtb CYP enzyme is most likely involved in the hydroxylation of the anti-tubercular drug SQ 109 (70). In a previous introduction, we mentioned that SQ 109 is a 1,2-ethylenediamine that targets MmpL 3 in Mtb (71). MmpL3 Is a mycolic acid transporter required for the incorporation of myacid into the Mtb cell wall. SQ 109 is in a phase II clinical trial for DS-TB, and SQ 109 has the best antimicrobial activity and the most promising toxicity (71). Bukhdruker et al. found that CYP124 will form a stable structure after binding to SQ 109 *in vitro* and hydroxylation. Therefore, they made the hypothesis that SQ109 is only a precursor and requires CYPs to participate in metabolism to produce anti-TB effects. This is the first time that CYPs in Mtb can biotransform anti-TB drugs. Assessment of the metabolism of anti-TB drug candidates using whole-cell-based systems or isolated Mtb enzymes may be a useful tool for anti-TB drug discovery.

### 3.4 Other anti-TB targets

#### 3.4.1 Iron metabolism

Iron is an essential element in life and a rare element in the human body. It is necessary for DNA replication, oxygen transport, energy generation, enzymatic redox reactions, and oxidative stress protection. Iron is also essential for the pathogenicity and virulence of Mtb. Siderophores play a very important role when Mtb uptakes iron from within host proteins. Mtb produces two aryl-cap siderophores, namely mystic (MBT) and carboxylic (cMBT), to chelate intracellular iron. Mycobacterium-expressed salicylic-AMP ligase (MbtA, encoded by the gene Rv2384) is an adenylylated enzyme that catalyzes the initial reaction of mycolin biosynthesis.MBTA has no human homolog and is required for the biosynthesis of salicylate-derived mystic (MBT) siderophore, a high-affinity Fe3 + chelator involved in iron (Fe) clearance and absorption, and a micronutrient critical for the growth and pathogenesis of Mtb. Are promising targets for the development of novel TB drugs that block siderophore biosynthesis. The 5 ′ -O- [N- (salicyl) amino-sulfonyl] adenosine (Sal-AMS) is specifically designed to inhibit the enzyme activity of MbtA and effectively block the growth and virulence of Mtb (72). Bythrow et al. (73) developed a salicylate adenylase inhibitor, salicyl-AMS and its derivatives, which can target MbtA, inhibit the biosynthesis of MBTs in Mtb, and more effectively limit Mtb growth under iron-limiting conditions. This study provides proof of principle for the druggability of salicylate adenylase, validates pharmacological inhibition of siderophore biosynthesis as a novel mechanism of antibiotic action, and establishes Sal-AMS as the first antimicrobial lead compound for the development of TB drugs targeting siderophore biosynthesis (73).

Iron-dependent repressor (IdeR) is a metal-sensing transcription factor that plays an important role in Mtb iron metabolism. It can regulate the free iron concentration in Mtb and is a central transcriptional regulator of the Mtb gene involved in Fe metabolism (74). IdeR can promote bacterial virulence, making it an important target in the therapeutic field (75). Crystallographic studies of IdeR revealed that only its dimeric structure has full functional activity to interact with conserved regions on DNA (76). Therefore, the development of its inhibitors is mainly by preventing the formation of dimers or blocking the interaction of IdeR and DNA-related sequences. Salimizand et al. (88) simulated the DBD of IdeR and DNA in the form of short peptides to target the binding site of whole protein and DNA operator and verified its potency through *in vitro* antibacterial experiments. The results showed the research value of the IdeR target and the potential of this protein as a drug target in the development of anti-TB drugs. However, there are no IdeR class inhibitors in preclinical studies.

#### 3.4.2 Immunotherapy of tuberculosis

Immune recognition, immune response, and immune regulation to Mtb determine the occurrence and development of TB (77). Since the body’s response to TB is based on innate immune cells and interactions between activated macrophages and specific T cells (78). Therefore, it is very feasible to enhance the protective immunity or regulate the adaptive immune response to promote the fight against tuberculosis bacilli. Anti-tuberculosis immunotherapy mainly includes activation of immune activity, enhancement of protective immunity, and suppression of adverse immune responses and inflammatory damage (79). TB immunotherapy drugs can effectively modulate the anti-TB immune response, provide new ways for the combined treatment of TB, and prevent and intervene in people at high risk of TB infection (80). This will enable TB treatment to achieve significant results and achieve the goal of “ultra-short-range chemotherapy.”

The clinical treatment difficulty of TB is not only the emergence of drug-resistant bacteria but also the immune evasion mechanism unique to Mtb. Resuscitation-promoting factor interacting protein (RipA) is a class of peptidoglycan hydrolyses, RipA is a key enzyme in TB cell division. Its absence leads to phenotypic abnormal growth defects, promoting the generation of IL-6, TNF- *α*, proinflammatory cytokines, rapid stimulation of MAPK and NF- *κ* B signaling to trigger the activation of dendritic cells (DC). Then induced the expression of the macrophage activation markers MHC-II, CD80, and CD86, promoted the Th 1 polarized immune response in naive CD4 T cells, increased proliferation, and activated T cells in Mtb-infected mice (81). Sung Jae Shin et al. (89) found that IFN- *γ* was secreted by RipA restimulation *in vitro* and RipA was fully recognized in the lungs and spleen of Mtb-infected mice. Furthermore, RipA induction induces initial CD4 + T cell proliferation, increased secretion of Th 1 cytokines, activation of splenic CD4 + T cells isolated from Mtb-infected mice, and subsequent inhibition of intracellular Mtb growth. And further validated the immunogenic function of RipA *in vivo*. With the help of the RipA-stimulated dendritic cells, the optimal T-cell activation was achieved to enhance the body’s anti-TB immune response. RipA has shown its research prospects as a subunit vaccine candidate for multiantigen TB.

The researchers found that peptidoglycan (PG) remodels through the action of several remodeling enzymes, such as transpeptidase, endopeptidase, carboxypeptidase, amidase, and transglycosylase. These enzymes have important roles in the hydrolysis, synthesis, crosslinking, and remodeling of PG and also mediate the repair of damaged cell wall material. Rv3717 (Ami 1) is a known mycobacterial amidase with the ability to cleave PG. Ami 1 synergistically with RipA, and Ami 1-deficient mutants are defective in survival in chronic infection in mice (82). Ami 1 and Ami 4 are involved in peptidoglycan degradation, recycling, and other processes during cell division, in which Ami 1 is thought to cooperate with RipA to safeguard Mtb division within host cells. It has been suggested that Ami 1 may promote the bacterial survival of mycobacteria within macrophages through the Ami 1-mediated reduction in host inflammation. These differences may be attributed to the induction of differential host immune responses within infected macrophages between nonpathogenic mycobacteria and pathogenic Mtb (82, 83). Reto Gulera Found that Ami 1 and Ami 4 depletion mediated the increased IFN *γ* independence of cytokines, which altered cell recruitment and activation during infection. Loss of Ami 1 is more immunoregulatory than Ami 4, with an increased pro-inflammatory immune response (84). Defective mutants can affect macrophages and host immune response *in vivo*, and Mtb Ami1 is an attractive target.

## 4 Summary

Although TB can be cured, the continuously increasing drug resistance of Mtb brings great pressure and challenges to the treatment of TB. To address the increasing burden of TB, there is an urgent need to improve treatment strategies, shorten treatment time, prevent the development of resistant strains, and study targeted agents for entirely new therapeutic mechanisms. With the completion of various whole genome sequencing of Mtb, the cracking of metabolic-related genes, the progress of biotechnology, and the progress of CADD, the research of anti-TB targets has developed rapidly, and many potential targets have been discovered. Through the deep exploration of the target genomics and drug resistance mechanisms, many novel compounds with potential anti-TB capabilities were selected. However, the screening, synthesis, and test of compounds are long and the cost is high, coupled with the different *in vitro* and *in vivo* experimental results. Therefore, there are not many compounds in the clinical trial stage. For the new compounds on the market in recent decades, only betaquoline is still the preferred drug. Here, we review the various kinds of targets that researchers have tried in recent years and summarize their inhibitors and inhibitory mechanisms. For drug-resistant TB, the switch to innovative targets and the development of novel scaffold compounds are thought to overcome drug-resistance problems. The application of CADD has greatly improved the discovery efficiency of novel anti-TB compounds, understood the mechanism of action, guided the structural optimization of lead compounds, and expected that better results can be obtained with the help of CADD. Complete integration of classical phenotypic screening and genomics approaches with proteomics-based protein analysis will help identify effective targets to develop safer and more effective drug candidates that can address the current therapeutic challenges of TB disease. The future belongs to those simple, short-range, highly specific, more sensitive, and effective solutions.

## Footnotes

^1^ www.specs.net

## References

[1] Alsayed SSR Gunosewoyo H. Tuberculosis: pathogenesis, current treatment regimens and new drug targets. Int J Mol Sci. (2023) 24:5202. doi: 10.3390/ijms24065202, PMID: 36982277 PMC10049048

[2] Bahuguna A Rawat DS. An overview of new antitubercular drugs, drug candidates, and their targets. Med Res Rev. (2020) 40:263–92. doi: 10.1002/med.21602 31254295

[3] Dass SA Balakrishnan V Arifin N Lim CSY Nordin F Tye GJ. The COVID-19/tuberculosis Syndemic and potential antibody therapy for TB based on the lessons learnt from the pandemic. Front Immunol. (2022) 13:833715. doi: 10.3389/fimmu.2022.833715 35242137 PMC8886238

[4] Shleider Carnero Canales C Marquez Cazorla J Furtado Torres AH Monteiro Filardi ET Di Filippo LD Costa PI. Advances in diagnostics and drug discovery against resistant and latent tuberculosis infection. Pharmaceutics. (2023) 15:2409. doi: 10.3390/pharmaceutics15102409, PMID: 37896169 PMC10610444

[5] Dartois V Dick T. Therapeutic developments for tuberculosis and nontuberculous mycobacterial lung disease. Nat Rev Drug Discov. (2024) 23:381–403. doi: 10.1038/s41573-024-00897-5, PMID: 38418662 PMC11078618

[6] Morrison H Jackson S McShane H. Controlled human infection models in COVID-19 and tuberculosis: current progress and future challenges. Front Immunol. (2023) 14:1211388. doi: 10.3389/fimmu.2023.1211388 37304270 PMC10248465

[7] Shariq M Sheikh JA Quadir N Sharma N Hasnain SE Ehtesham NZ. COVID-19 and tuberculosis: the double whammy of respiratory pathogens. Eur Respir Rev. (2022) 31:210264. doi: 10.1183/16000617.0264-2021 35418488 PMC9488123

[8] Kant S Tyagi R. The impact of COVID-19 on tuberculosis: challenges and opportunities. Ther Adv Infect Dis. (2021) 8:20499361211016973. doi: 10.1177/20499361211016973 34178322 PMC8193657

[9] Hopewell PC Reichman LB Castro KG. Parallels and mutual lessons in tuberculosis and COVID-19 transmission, prevention, and control. Emerg Infect Dis. (2021) 27:681–6. doi: 10.3201/eid2703.203456, PMID: 33213689 PMC7920655

[10] Suarez I Funger SM Kroger S Rademacher J Fatkenheuer G Rybniker J. The diagnosis and treatment of tuberculosis. Dtsch Arztebl Int. (2019) 116:729–35. doi: 10.3238/arztebl.2019.0729, PMID: 31755407

[11] Peloquin CA Davies GR. The treatment of tuberculosis. Clin Pharmacol Ther. (2021) 110:1455–66. doi: 10.1002/cpt.2261 33837535

[12] Yang L Hu X Chai X Ye Q Pang J Li D: Opportunities for overcoming tuberculosis: Emerging targets and their inhibitors. Drug Discov Today (2022), 27:326–336 34537334 10.1016/j.drudis.2021.09.003

[13] Conradie F Bagdasaryan TR Borisov S Howell P Mikiashvili L Ngubane N. Bedaquiline-Pretomanid-linezolid regimens for drug-resistant tuberculosis. N Engl J Med. (2022) 387:810–23. doi: 10.1056/NEJMoa2119430 36053506 PMC9490302

[14] Cole ST Brosch R Parkhill J Garnier T Churcher C Harris D. Deciphering the biology of *Mycobacterium tuberculosis* from the complete genome sequence. Nature. (1998) 393:537–44. doi: 10.1038/31159, PMID: 9634230

[15] Canales CSC Pavan AR Dos Santos JL Pavan FR. In silico drug design strategies for discovering novel tuberculosis therapeutics. Expert Opin Drug Discov. (2024) 19:471–91. doi: 10.1080/17460441.2024.2319042, PMID: 38374606

[16] Amandy FV Neri GLL Manzano JAH Go AD Macabeo APG. Polypharmacology-driven discovery and Design of Highly Selective, dual and multitargeting inhibitors of *Mycobacterium tuberculosis* - a review. Curr Drug Targets. (2024) 25:620–34. doi: 10.2174/0113894501306302240526160804 38859782

[17] Kuang W Zhang H Wang X Yang P. Overcoming *Mycobacterium tuberculosis* through small molecule inhibitors to break down cell wall synthesis. Acta Pharm Sin B. (2022) 12:3201–14. doi: 10.1016/j.apsb.2022.04.014, PMID: 35967276 PMC9366312

[18] Capela R Felix R Clariano M Nunes D Perry MJ Lopes F. Target identification in anti-tuberculosis drug discovery. Int J Mol Sci. (2023) 24:482. doi: 10.3390/ijms241310482, PMID: 37445660 PMC10341898

[19] Meyer FM Bramkamp M. Cell wall synthesizing complexes in Mycobacteriales. Curr Opin Microbiol. (2024) 79:102478. doi: 10.1016/j.mib.2024.102478, PMID: 38653035

[20] Xu X Dong B Peng L Gao C He Z Wang C. Anti-tuberculosis drug development via targeting the cell envelope of *Mycobacterium tuberculosis*. Front Microbiol. (2022) 13:1056608. doi: 10.3389/fmicb.2022.1056608, PMID: 36620019 PMC9810820

[21] Sreelatha S Nagarajan U Natarajan S. Protein targets in Mycobacterium tuberculosis and their inhibitors for therapeutic implications: a narrative review. Int J Biol Macromol. (2023) 243:125022. doi: 10.1016/j.ijbiomac.2023.125022 37244342

[22] Chen K Xu R Hu X Li D Hou T Kang Y. Recent advances in the development of DprE1 inhibitors using AI/CADD approaches. Drug Discov Today. (2024) 29:103987. doi: 10.1016/j.drudis.2024.103987, PMID: 38670256

[23] Yadav S Soni A Tanwar O Bhadane R Besra GS Kawathekar N. DprE1 inhibitors: enduring aspirations for future Antituberculosis drug discovery. ChemMedChem. (2023) 18:e202300099. doi: 10.1002/cmdc.202300099, PMID: 37246503

[24] Huszar S Chibale K Singh V. The quest for the holy grail: new antitubercular chemical entities, targets and strategies. Drug Discov Today. (2020) 25:772–80. doi: 10.1016/j.drudis.2020.02.003, PMID: 32062007 PMC7215093

[25] Makarov V Salina E Reynolds RC Kyaw Zin PP Ekins S. Molecule property analyses of active compounds for *Mycobacterium tuberculosis*. J Med Chem. (2020) 63:8917–55. doi: 10.1021/acs.jmedchem.9b02075, PMID: 32259446 PMC7702311

[26] Oh S Trifonov L Yadav VD Barry CE 3rd Boshoff HI. Tuberculosis drug discovery: a decade of hit assessment for defined targets. Front Cell Infect Microbiol. (2021) 11:611304. doi: 10.3389/fcimb.2021.611304, PMID: 33791235 PMC8005628

[27] Dube PS Legoabe LJ Jordaan A Sigauke L Warner DF Beteck RM. Quinolone analogues of benzothiazinone: synthesis, antitubercular structure-activity relationship and ADME profiling. Eur J Med Chem. (2023) 258:115539. doi: 10.1016/j.ejmech.2023.115539, PMID: 37321107

[28] Piton J Vocat A Lupien A Foo CS Riabova O Makarov V. Structure-based drug design and characterization of sulfonyl-Piperazine Benzothiazinone inhibitors of DprE1 from *Mycobacterium tuberculosis*. Antimicrob Agents Chemother. (2018) 62:681. doi: 10.1128/AAC.00681-18, PMID: 30012754 PMC6153800

[29] Amado PSM Woodley C Cristiano MLS O’Neill PM. Recent advances of DprE1 inhibitors against *Mycobacterium tuberculosis*: computational analysis of physicochemical and ADMET properties. ACS Omega. (2022) 7:40659–81. doi: 10.1021/acsomega.2c05307, PMID: 36406587 PMC9670723

[30] Finger V Kufa M Soukup O Castagnolo D Roh J Korabecny J. Pyrimidine derivatives with antitubercular activity. Eur J Med Chem. (2023) 246:114946. doi: 10.1016/j.ejmech.2022.114946 36459759

[31] Kb S Kumari A Shetty D Fernandes E Dv C Jays J. Structure based pharmacophore modelling approach for the design of azaindole derivatives as DprE1 inhibitors for tuberculosis. J Mol Graph Model. (2020) 101:107718. doi: 10.1016/j.jmgm.2020.107718 32949960

[32] North EJ Schwartz CP Zgurskaya HI Jackson M. Recent advances in mycobacterial membrane protein large 3 inhibitor drug design for mycobacterial infections. Expert Opin Drug Discov. (2023) 18:707–24. doi: 10.1080/17460441.2023.2218082 37226498 PMC10330604

[33] Sethiya JP Sowards MA Jackson M North EJ. MmpL3 inhibition: a new approach to treat nontuberculous mycobacterial infections. Int J Mol Sci. (2020) 21:6202. doi: 10.3390/ijms21176202, PMID: 32867307 PMC7503588

[34] Umare MD Khedekar PB Chikhale RV. Mycobacterial membrane protein large 3 (MmpL3) inhibitors: a promising approach to combat tuberculosis. ChemMedChem. (2021) 16:3136–48. doi: 10.1002/cmdc.202100359, PMID: 34288519

[35] Carbone J Paradis NJ Bennet L Alesiani MC Hausman KR Wu C. Inhibition mechanism of anti-TB drug SQ109: allosteric inhibition of TMM translocation of *Mycobacterium Tuberculosis* MmpL3 transporter. J Chem Inf Model. (2023) 63:5356–74. doi: 10.1021/acs.jcim.3c00616, PMID: 37589273 PMC10466384

[36] Heinrich N Dawson R du Bois J Narunsky K Horwith G Phipps AJ. Early phase evaluation of SQ109 alone and in combination with rifampicin in pulmonary TB patients. J Antimicrob Chemother. (2015) 70:1558–66. doi: 10.1093/jac/dku553, PMID: 25630641

[37] Imran M Arora MK Chaudhary A Khan SA Kamal M Alshammari MM. MmpL3 inhibition as a promising approach to develop novel therapies against tuberculosis: a spotlight on SQ109, clinical studies, and patents literature. Biomedicines. (2022) 10:2793. doi: 10.3390/biomedicines10112793, PMID: 36359313 PMC9687596

[38] Ge Y Luo Q Liu L Shi Q Zhang Z Yue X. S288T mutation altering MmpL3 periplasmic domain channel and H-bond network: a novel dual drug resistance mechanism. J Mol Model. (2024) 30:39. doi: 10.1007/s00894-023-05814-y, PMID: 38224406

[39] Qu H Xin Y Dong X Ma Y. An rmlA gene encoding d-glucose-1-phosphate thymidylyltransferase is essential for mycobacterial growth. FEMS Microbiol Lett. (2007) 275:237–43. doi: 10.1111/j.1574-6968.2007.00890.x, PMID: 17784859

[40] Qu D Zhao X Sun Y Wu FL Tao SC. *Mycobacterium tuberculosis* Thymidylyltransferase RmlA is negatively regulated by Ser/Thr protein kinase PknB. Front Microbiol. (2021) 12:643951. doi: 10.3389/fmicb.2021.643951, PMID: 33868202 PMC8044546

[41] Dhaked DK Bala Divya M Guruprasad L. A structural and functional perspective on the enzymes of *Mycobacterium tuberculosis* involved in the L-rhamnose biosynthesis pathway. Prog Biophys Mol Biol. (2019) 145:52–64. doi: 10.1016/j.pbiomolbio.2018.12.004, PMID: 30550737

[42] Prasad MS Bhole RP Khedekar PB Chikhale RV. Mycobacterium enoyl acyl carrier protein reductase (InhA): a key target for antitubercular drug discovery. Bioorg Chem. (2021) 115:105242. doi: 10.1016/j.bioorg.2021.105242 34392175

[43] Wahan SK Bhargava G Chawla V Chawla PA. Unlocking InhA: novel approaches to inhibit *Mycobacterium tuberculosis*. Bioorg Chem. (2024) 146:107250. doi: 10.1016/j.bioorg.2024.107250, PMID: 38460337

[44] Alcaraz M Roquet-Baneres F Leon-Icaza SA Abendroth J Boudehen YM Cougoule C. Efficacy and mode of action of a direct inhibitor of *Mycobacterium abscessus* InhA. ACS Infect Dis. (2022) 8:2171–86. doi: 10.1021/acsinfecdis.2c00314, PMID: 36107992

[45] Batson S de Chiara C Majce V Lloyd AJ Gobec S Rea D. Inhibition of D-ala:D-ala ligase through a phosphorylated form of the antibiotic D-cycloserine. Nat Commun. (2017) 8:1939. doi: 10.1038/s41467-017-02118-7, PMID: 29208891 PMC5717164

[46] Yang S Xu Y Wang Y Ren F Li S Ding W. The biological properties and potential interacting proteins of d-Alanyl-d-alanine ligase a from *Mycobacterium tuberculosis*. Molecules. (2018) 23:324. doi: 10.3390/molecules23020324 29401644 PMC6017538

[47] Qin Y Xu L Teng Y Wang Y Ma P. Discovery of novel antibacterial agents: recent developments in D-alanyl-D-alanine ligase inhibitors. Chem Biol Drug Des. (2021) 98:305–22. doi: 10.1111/cbdd.13899 34047462

[48] Khan T Sankhe K Suvarna V Sherje A Patel K Dravyakar B. DNA gyrase inhibitors: Progress and synthesis of potent compounds as antibacterial agents. Biomed Pharmacother. (2018) 103:923–38. doi: 10.1016/j.biopha.2018.04.021 29710509

[49] Pakeeraiah K Mal S Mahapatra M Mekap SK Sahu PK Paidesetty SK. Schematic-portfolio of potent anti-microbial scaffolds targeting DNA gyrase: unlocking ways to overcome resistance. Int J Biol Macromol. (2024) 256:128402. doi: 10.1016/j.ijbiomac.2023.128402, PMID: 38035955

[50] Salman M Sharma P Kumar M Ethayathulla AS Kaur P. Targeting novel sites in DNA gyrase for development of anti-microbials. Brief Funct Genomics. (2023) 22:180–94. doi: 10.1093/bfgp/elac029, PMID: 36064602

[51] Pakamwong B Thongdee P Kamsri B Phusi N Kamsri P Punkvang A. Identification of potent DNA gyrase inhibitors active against Mycobacterium tuberculosis. J Chem Inf Model. (2022) 62:1680–90. doi: 10.1021/acs.jcim.1c01390, PMID: 35347987

[52] Monama MZ Olotu F Tastan Bishop O. Investigation of multi-subunit *Mycobacterium tuberculosis* DNA-directed RNA polymerase and its rifampicin resistant mutants. Int J Mol Sci. (2023) 24:3313. doi: 10.3390/ijms24043313, PMID: 36834726 PMC9965755

[53] Lin WH Lee WT Tsai HY Jou R. Disputed rpoB mutations in Mycobacterium tuberculosis and tuberculosis treatment outcomes. Antimicrob Agents Chemother. (2021) 65:e0157320. doi: 10.1128/AAC.01573-20, PMID: 33846134 PMC8218645

[54] Chakravorty S Simmons AM Rowneki M Parmar H Cao Y Ryan J. The new Xpert MTB/RIF ultra: improving detection ofMycobacterium tuberculosisand resistance to rifampin in an assay suitable for point-of-care testing. MBio. (2017) 8:17. 10.1128/mBio.00812-17 PMC5574709 28851844

[55] Loukil A Kirtania P Bedotto M Drancourt M. FISHing *Mycobacterium tuberculosis* complex by use of a rpoB DNA probe bait. J Clin Microbiol. (2018) 56:10–1128. doi: 10.1128/JCM.00568-18, PMID: 30068538 PMC6156301

[56] Mann L Ganapathy US Abdelaziz R Lang M Zimmerman MD Dartois V. In vitro profiling of the synthetic RNA polymerase inhibitor MMV688845 against *Mycobacterium abscessus*. Microbiol Spectr. (2022) 10:e0276022. doi: 10.1128/spectrum.02760-22 36377951 PMC9769904

[57] Jian Y Merceron R De Munck S Forbes HE Hulpia F Risseeuw MDP. Endeavors towards transformation of *M. tuberculosis* thymidylate kinase (MtbTMPK) inhibitors into potential antimycobacterial agents. Eur J Med Chem. (2020) 206:112659. doi: 10.1016/j.ejmech.2020.112659, PMID: 32823003 PMC11000207

[58] Venugopala KN Tratrat C Pillay M Chandrashekharappa S Al-Attraqchi OHA Aldhubiab BE. In silico design and synthesis of Tetrahydropyrimidinones and Tetrahydropyrimidinethiones as potential thymidylate kinase inhibitors exerting anti-TB activity against *Mycobacterium tuberculosis*. Drug Des Devel Ther. (2020) 14:1027–39. doi: 10.2147/DDDT.S228381, PMID: 32214795 PMC7082623

[59] Dhulap A Banerjee P. ATP synthase, an emerging target in TB drug discovery: review of SAR and clinical pharmacology of Diarylquinoline inhibitors. Curr Drug Targets. (2021) 22:1207–21. doi: 10.2174/1389450122666210122084332, PMID: 33480344

[60] Tantry SJ Markad SD Shinde V Bhat J Balakrishnan G Gupta AK. Discovery of Imidazo[1,2-a]pyridine ethers and Squaramides as selective and potent inhibitors of mycobacterial adenosine triphosphate (ATP) synthesis. J Med Chem. (2017) 60:1379–99. doi: 10.1021/acs.jmedchem.6b01358, PMID: 28075132

[61] Singh S Roy KK Khan SR Kashyap VK Sharma A Jaiswal S. Novel, potent, orally bioavailable and selective mycobacterial ATP synthase inhibitors that demonstrated activity against both replicating and non-replicating *M. tuberculosis*. Bioorg Med Chem. (2015) 23:742–52. doi: 10.1016/j.bmc.2014.12.060, PMID: 25614114

[62] Khan SR Singh S Roy KK Akhtar MS Saxena AK Krishnan MY. Biological evaluation of novel substituted chloroquinolines targeting mycobacterial ATP synthase. Int J Antimicrob Agents. (2013) 41:41–6. doi: 10.1016/j.ijantimicag.2012.09.012 23141113

[63] Saha P Das S Indurthi HK Kumar R Roy A Kalia NP. Cytochrome bd oxidase: an emerging anti-tubercular drug target. RSC Med Chem. (2024) 15:769–87. doi: 10.1039/D3MD00587A, PMID: 38516593 PMC10953478

[64] Harikishore A Mathiyazakan V Pethe K Gruber G. Novel targets and inhibitors of the *Mycobacterium tuberculosis* cytochrome bd oxidase to foster anti-tuberculosis drug discovery. Expert Opin Drug Discov. (2023) 18:917–27. doi: 10.1080/17460441.2023.2224553, PMID: 37332221

[65] de Jager VR Dawson R van Niekerk C Hutchings J Kim J Vanker N. Telacebec (Q203), a new antituberculosis agent. N Engl J Med. (2020) 382:1280–1. doi: 10.1056/NEJMc1913327 32212527

[66] Lu P Asseri AH Kremer M Maaskant J Ummels R Lill H. The anti-mycobacterial activity of the cytochrome bcc inhibitor Q203 can be enhanced by small-molecule inhibition of cytochrome bd. Sci Rep. (2018) 8:2625. doi: 10.1038/s41598-018-20989-8, PMID: 29422632 PMC5805707

[67] Kalia NP Hasenoehrl EJ Ab Rahman NB Koh VH Ang MLT Sajorda DR. Exploiting the synthetic lethality between terminal respiratory oxidases to kill Mycobacterium tuberculosis and clear host infection. Proc Natl Acad Sci USA. (2017) 114:7426–31. doi: 10.1073/pnas.1706139114, PMID: 28652330 PMC5514758

[68] Chilamakuru NB Vn AD VB G Pallaprolu N Dande A Nair D. New synergistic benzoquinone scaffolds as inhibitors of mycobacterial cytochrome bc1 complex to treat multi-drug resistant tuberculosis. Eur J Med Chem. (2024) 272:116479. doi: 10.1016/j.ejmech.2024.116479, PMID: 38733886

[69] Manikandan P Nagini S. Cytochrome P450 structure, function and clinical significance: a review. Curr Drug Targets. (2018) 19:38–54. doi: 10.2174/1389450118666170125144557 28124606

[70] Sacksteder KA Protopopova M Barry CE 3rd Andries K Nacy CA. Discovery and development of SQ109: a new antitubercular drug with a novel mechanism of action. Future Microbiol. (2012) 7:823–37. doi: 10.2217/fmb.12.56, PMID: 22827305 PMC3480206

[71] Malwal SR Zimmerman MD Alvarez N Sarathy JP Dartois V Nacy CA. Structure, in vivo detection, and antibacterial activity of metabolites of SQ109, an anti-infective drug candidate. ACS Infect Dis. (2021) 7:2492–507. doi: 10.1021/acsinfecdis.1c00259, PMID: 34279904 PMC8918016

[72] Qiao C Gupte A Boshoff HI Wilson DJ Bennett EM Somu RV. 5’-O-[(N-acyl)sulfamoyl]adenosines as antitubercular agents that inhibit MbtA: an adenylation enzyme required for siderophore biosynthesis of the mycobactins. J Med Chem. (2007) 50:6080–94. doi: 10.1021/jm070905o, PMID: 17967002 PMC2539069

[73] Bythrow GV Mohandas P Guney T Standke LC Germain GA Lu X. Kinetic analyses of the siderophore biosynthesis inhibitor Salicyl-AMS and analogues as MbtA inhibitors and antimycobacterial agents. Biochemistry. (2019) 58:833–47. doi: 10.1021/acs.biochem.8b01153 30582694 PMC6530907

[74] Kurthkoti K Tare P Paitchowdhury R Gowthami VN Garcia MJ Colangeli R. The mycobacterial iron-dependent regulator IdeR induces ferritin (bfrB) by alleviating Lsr2 repression. Mol Microbiol. (2015) 98:864–77. doi: 10.1111/mmi.13166, PMID: 26268801 PMC4879814

[75] Ghosh S Chandra N Vishveshwara S. Mechanism of Iron-dependent repressor (IdeR) activation and DNA binding: a molecular dynamics and protein structure network study. PLoS Comput Biol. (2015) 11:e1004500. doi: 10.1371/journal.pcbi.1004500, PMID: 26699663 PMC4689551

[76] Wisedchaisri G Chou CJ Wu M Roach C Rice AE Holmes RK. Crystal structures, metal activation, and DNA-binding properties of two-domain IdeR from *Mycobacterium tuberculosis*. Biochemistry. (2007) 46:436–47. doi: 10.1021/bi0609826 17209554

[77] Roy A Kumari Agnivesh P Sau S Kumar S Pal Kalia N. Tweaking host immune responses for novel therapeutic approaches against *Mycobacterium tuberculosis*. Drug Discov Today. (2023) 28:103693. doi: 10.1016/j.drudis.2023.103693, PMID: 37390961

[78] Ahmad F Rani A Alam A Zarin S Pandey S Singh H. Macrophage: a cell with many faces and functions in tuberculosis. Front Immunol. (2022) 13:747799. doi: 10.3389/fimmu.2022.747799, PMID: 35603185 PMC9122124

[79] Ernst JD. Mechanisms of *M. tuberculosis* immune evasion as challenges to TB vaccine design. Cell Host Microbe. (2018) 24:34–42. doi: 10.1016/j.chom.2018.06.004, PMID: 30001523 PMC6482466

[80] Mi J Liang Y Liang J Gong W Wang S Zhang J. The research Progress in immunotherapy of tuberculosis. Front Cell Infect Microbiol. (2021) 11:763591. doi: 10.3389/fcimb.2021.763591, PMID: 34869066 PMC8634162

[81] Healy C Gouzy A Ehrt S. Peptidoglycan hydrolases RipA and Ami1 are critical for replication and persistence of *Mycobacterium tuberculosis* in the host. MBio. (2020) 11:10–1128. doi: 10.1128/mBio.03315-19 PMC7064781 32127458

[82] Gong Z Yang W Zhang H Xiang X Zeng J Han S. *Mycobacterium tuberculosis* Rv3717 enhances the survival of *Mycolicibacterium smegmatis* by inhibiting host innate immune and caspase-dependent apoptosis. Infect Genet Evol. (2020) 84:104412. doi: 10.1016/j.meegid.2020.104412 32531516

[83] Madhvi A Mishra H Chegou NN Tromp G Van Heerden CJ Pietersen RD. Distinct host-immune response toward species related intracellular mycobacterial killing: a transcriptomic study. Virulence. (2020) 11:170–82. doi: 10.1080/21505594.2020.1726561, PMID: 32052695 PMC7051142

[84] Kieswetter NS Ozturk M Jones SS Senzani S Chengalroyen MD Brombacher F. Deletion of N-acetylmuramyl-L-alanine amidases alters the host immune response to *Mycobacterium tuberculosis* infection. Virulence. (2021) 12:1227–38. doi: 10.1080/21505594.2021.1914448, PMID: 33980132 PMC8128173

[85] Persing D Kwiatkowski R Jones M Alland D. The New Xpert MTB/RIF Ultra: Improving Detection of Mycobacterium tuberculosis and Resistance to Rifampin in an Assay Suitable for Point-of-Care Testing. mBio. (2017) 8:e00812–17. doi: 10.1128/mBio.00812-17 28851844 PMC5574709

[86] El-Shoukrofy MS Atta A Fahmy S Sriram D Mahran MA Labouta IM. New tetrahydropyrimidine-1,2,3-triazole clubbed compounds: Antitubercular activity and Thymidine Monophosphate Kinase (TMPKmt) inhibition. Bioorg Chem. (2023) 131:106312. doi: 10.1016/j.bioorg.2022.106312 36528922

[87] Bukhdruker S Varaksa T Grabovec I Marin E Shabunya P Kadukova M. Hydroxylation of Antitubercular Drug Candidate, SQ109, by Mycobacterial Cytochrome P450. Int J Mol Sci. (2020) 21:7683. doi: 10.3390/ijms21207683 33081390 PMC7589583

[88] Kwon KW Choi HG Kim KS Park SA Kim HJ Shin SJ. BCG-booster vaccination with HSP90-ESAT-6-HspX-RipA multivalent subunit vaccine confers durable protection against hypervirulent Mtb in mice. NPJ Vaccines. (2024) 9:55. doi: 10.1038/s41541-024-00847-7 38459038 PMC10923817

[89] Salimizand H Jamehdar SA Nik LB Sadeghian H. Design of peptides interfering with iron-dependent regulator (IdeR) and evaluation of Mycobacterium tuberculosis growth inhibition. Iran J Basic Med Sci. (2017) 20:722–728. doi: 10.22038/IJBMS.2017.8859 28868128 PMC5569447

